# PPARdelta: A key modulator in the pathogenesis of diabetes mellitus and *Mycobacterium tuberculosis* co-morbidity

**DOI:** 10.1016/j.isci.2024.110046

**Published:** 2024-05-22

**Authors:** Halemah AlSaeed, Mohammed J.A. Haider, Fawaz Alzaid, Fahd Al-Mulla, Rasheed Ahmad, Fatema Al-Rashed

**Affiliations:** 1 Immunology and Microbiology Department, Dasman Diabetes Institute, Al-Soor Street, Dasman, Kuwait, PO BOX 1180, Dasman 15462, State of Kuwait; 2 Department of Biological Sciences, Faculty of Science, Kuwait University, PO BOX 5969, Safat 13060, State of Kuwait; 3 Bioenergetics Department, Dasman Diabetes Institute, Kuwait City 15462, Kuwait; 4 Université Paris Cité, INSERM UMR-S1151, CNRS UMR-S8253, Institut Necker Enfants Malades, 75015 Paris, France; 5 Genetics and Bioinformatics Department, Dasman Diabetes Institute, Kuwait, Kuwait

**Keywords:** Human Physiology, Human metabolism, Immunology, Bacteriology, Molecular microbiology

## Abstract

The interplay between lipid metabolism and immune response in macrophages plays a pivotal role in various infectious diseases, notably tuberculosis (TB). Herein, we illuminate the modulatory effect of heat-killed *Mycobacterium tuberculosis* (HKMT) on macrophage lipid metabolism and its implications on the inflammatory cascade. Our findings demonstrate that HKMT potently activates the lipid scavenger receptor, CD36, instigating lipid accumulation. While CD36 inhibition mitigated lipid increase, it unexpectedly exacerbated the inflammatory response. Intriguingly, this paradoxical effect was linked to an upregulation of PPARδ. Functional analyses employing PPARδ modulation revealed its central role in regulating both lipid dynamics and inflammation, suggesting it as a potential therapeutic target. Moreover, primary monocytic cells from diabetic individuals, a demographic at amplified risk of TB, exhibited heightened PPARδ expression and inflammation, further underscoring its pathological relevance. Targeting PPARδ in these cells effectively dampened the inflammatory response, offering a promising therapeutic avenue against TB.

## Highlights

• Monocytic cells influence TB infection and fat metabolism
• HKMT’s activation of CD36 leads to monocytic cells lipid accumulation
• PPARδ modulates lipid dynamics and inflammation independent of CD36 inhibition
• Diabetics exhibit heightened PPARδ expression, linking to increased TB risk

## Graphical abstract

**Figure:**
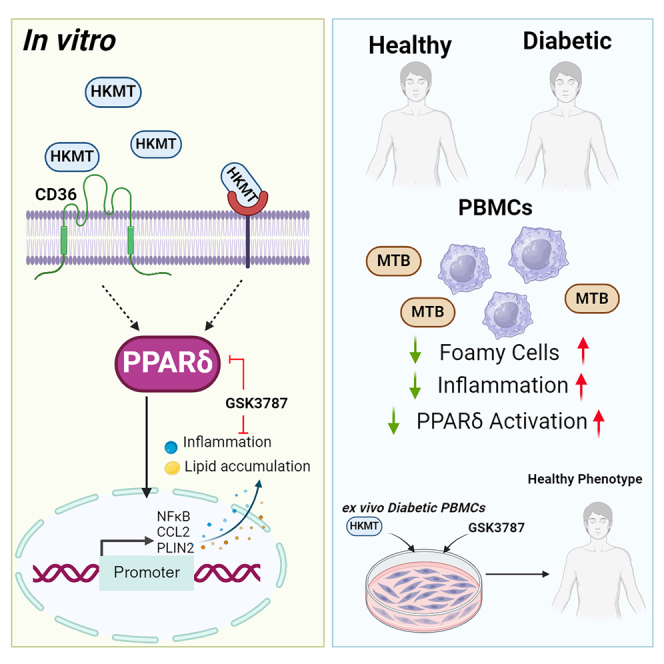

## Introduction

Tuberculosis (TB), caused by *Mycobacterium tuberculosis* (MTB), remains one of the leading infectious diseases worldwide, with a substantial global morbidity and mortality rate.^1^ Diabetes mellitus (DM), a metabolic disorder characterized by chronic hyperglycemia, has been identified as a significant risk factor for TB, with diabetic patients showing increased susceptibility to TB infection and poorer treatment outcomes.^2^^,^^3^ The intricate relationship between these two conditions, often termed as “twin epidemics”, has prompted intense research to understand the underlying molecular mechanisms that facilitate this co-morbidity.^4^

One of the hallmarks of MTB infection is its ability to modulate host lipid metabolism, leading to the accumulation of lipids within infected macrophages. This lipid-rich environment is believed to support bacterial persistence and provide a shield against host immune responses.^5^^,^^6^ As macrophages are considered the primary host cells for MTB, they undergo a transformation into “foamy” cells characterized by lipid droplet accumulation upon MTB infection.^7^ These lipid-laden macrophages play a dual role: they provide a nutrient-rich environment for the residing bacteria and simultaneously become less effective in antimicrobial functions, thus facilitating bacterial survival and persistence.^8^

Central to this process of lipid accumulation is CD36, a scavenger receptor and fatty acid translocase. CD36 is intricately involved in the uptake of oxidized low-density lipoproteins (oxLDLs) and long-chain fatty acids.^9^ Recent studies have illuminated the role of CD36 in MTB-infected macrophages, suggesting that CD36-mediated lipid uptake is a pivotal factor in the transformation of macrophages into a foamy phenotype.^10^ Furthermore, CD36 has been implicated in recognizing and binding to MTB, thereby modulating the host immune response and influencing the course of the disease.^11^

Despite the central role of CD36 in macrophage foaming and its evident involvement in MTB infection, attempts to leverage CD36 as a therapeutic target have been largely unsuccessful.^12^^,^^13^ This underscores a potential gap in our understanding of the intricate mechanisms governing the interaction of CD36 with MTB. While CD36 is pivotal in lipid uptake and the formation of foamy macrophages, it might also be part of a larger network of molecular players that collectively modulate the host response to MTB.^14^^,^^15^ Among such molecular players, the peroxisome proliferator-activated receptors (PPARs) have emerged as a crucial modulator in both lipid metabolism and inflammatory processes up-stream of CD36, with potential implications in the pathogenesis of both DM and TB.^16^^,^^17^ However, the impact of the PPARs family in the context of diabetes and tuberculosis comorbidity has not been thoroughly investigated.

In this study, we report a significant role for PPARδ in exacerbation of lipid accumulation and inflammation in *in vitro* and *ex vivo* models of tuberculosis. Using commercially available heat-killed preparations of *M. tuberculosis* (HKMT) derived from the avirulent strain H37Ra, we show that HKMT induces inflammation in THP-1 cells and concomitant lipid accumulation through a PPARδ-CD36 axis. Importantly, this phenotype is abrogated upon using pharmacological inhibitors and siRNA constructs. Furthermore, RT-qPCR analysis reveals upregulation of PPARδ gene expression in monocytes isolated from diabetics compared with non-diabetics, and this is paralleled by increased CD36 and CCL2 gene expression, thereby indicative of increased inflammation and lipid uptake. Further, correlation analysis reveals a strong association between the expression of PPARδ and worsening of glucose homeostasis markers (HOMA-IR and HbA1C). Additionally, using peripheral blood mononuclear cells (PBMCs) from patients with or without diabetes, we show that pharmacological inhibition of PPARδ, but not CD36, mitigates HKMT-induced inflammatory responses. Together, our data provide novel insight into the mechanistic basis underlying diabetes-tuberculosis comorbidity and shed light on PPARδ as a potential target for therapeutic intervention.

## Results

### HKMT induces lipid accumulation and inflammation in a dose-dependent manner

**Figure 1:**
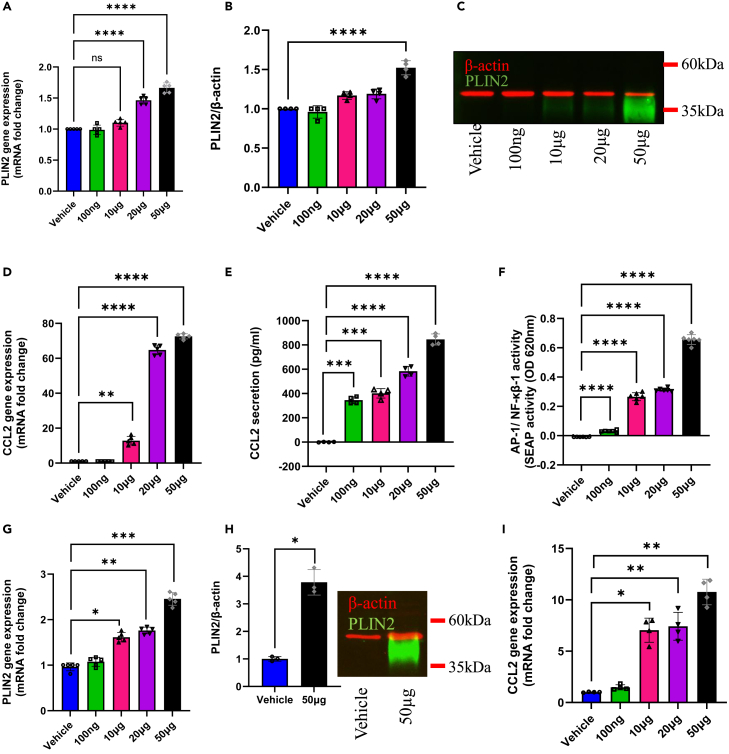
HKMT induces lipid accumulation and inflammation in THP-1 cells in a dose-dependent manner THP-1 cells were treated with several doses of HKMT (0, 100 ng, 10 μg, 20 μg, and 50 μg). Cells were harvested for total mRNA or total protein extraction as described in STAR Methods. (A) Gene expression of PLIN2 was determined using RT-qPCR. (B) Protein expression of PLIN2 corrected over β-actin (C) Representative multiplex western blot showing the effect of HKMT dose response on PLIN2 expression. (D) Gene expression of CCL2 determined by RT-qPCR (E) Analysis of secreted inflammatory mediator CCL2 by ELISA. (F) NF-κB reporter monocytic cells were treated with several doses of HKMT (0, 100 ng, 10 μg, 20 μg, and 50 μg). Cell culture media were assayed for SEAP reporter activity (degree of NF-κB activation). For *ex vivo* work, isolated PBMCs were cultured as previously described and pre-treated with several doses of HKMT (0, 100 ng, 10 μg, 20 μg, and 50 μg). (G) Gene expression of PLIN2 was determined using RT-qPCR (H) Protein expression of PLIN2 corrected over β-actin. (I) Gene expression of CCL2 determined by RT-qPCR. Data are presented as mean ± SEM values (*n* = 3–4, biological replicates). One-way ANOVA with Tukey’s multiple comparisons test was used to compare between more than two groups, while Student’s t test was used to compare the means between two groups. ∗*p* < 0.05, ∗∗*p* < 0.01, ∗∗∗*p* < 0.001, ∗∗∗∗*p* < 0.0001.

The remarkable capacity of MTB to modulate the delicate balance of host lipid regulation has garnered significant attention from multiple research groups. This process is believed to be crucial for pathogen survival because foamy macrophages serve as a primary environment for pathogen replication, inflammation induction, and TB development.^18^ To investigate whether HKMT exhibits similar effects, THP1 cells were exposed to varying doses of HKMT and the extent of lipid accumulation within these cells was examined. Remarkably, HKMT displayed a dose-dependent augmentation of perilipin-2 (PLIN2), the protein that surrounds lipid droplets at both the mRNA and protein levels (Figures 1A–1C). These findings suggest that HKMT has a pronounced impact on lipid metabolism and storage in THP-1 cells. Furthermore, this effect extended to the secretion of inflammatory cytokines, as demonstrated by the dose-dependent elevation of CCL2 gene (Figure 1D) and protein expression (Figure 1E). The impact of HKMT was further elucidated by its ability to enhance NF-κB/AP-1 activity in human monocytic reporter cells, with this induction also displaying a clear dose-dependent pattern (Figure 1F). In addition to the THP-1 cell model, the effect of HKMT on lipid metabolism and storage was thoroughly examined using an *ex vivo* model with freshly isolated PBMCs from healthy donors. Consistent with the THP-1 cell findings, a dose-dependent increase in PLIN2 gene expression was observed in this model as well (Figure 1G), along with a significant elevation in PLIN2 protein levels when comparing non-treated vehicle control to cells treated with 50 μg HKMT (Figure 1H). Additionally, a similar pattern was seen in the dose-dependent induction of CCL2 gene expression (Figure 1I). Together, these results indicate that HKMT triggers an enhanced inflammatory response in a manner similar to that observed for MTB. Importantly, this can potentially contribute to the progression of TB pathology. It also suggests that HKMT has the capacity to activate key transcription factors involved in inflammation and immune responses.

### HKMT induces lipid accumulation through the activation of CD36 scavenger protein

**Figure 2:**
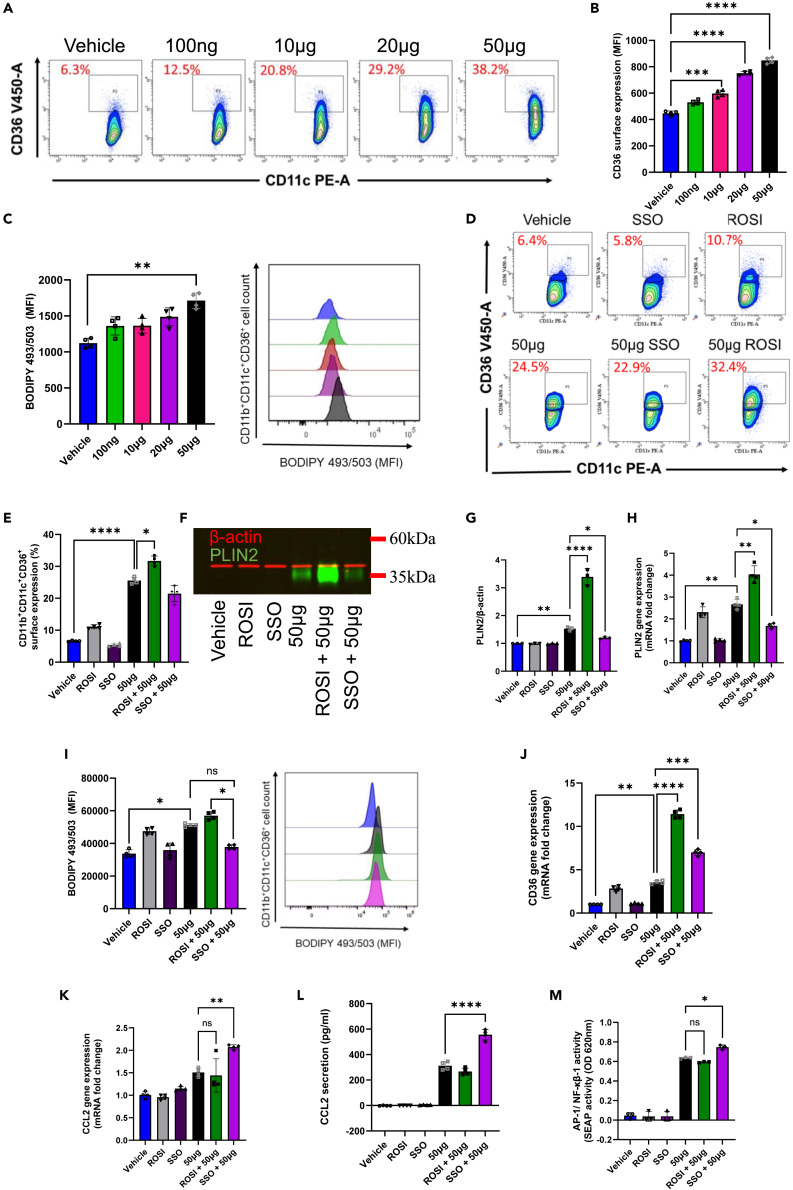
HKMT induces lipid accumulation through the activation of CD36 scavenger protein THP-1 cells were treated with several doses of HKMT (0, 100 ng, 10 μg, 20 μg, and 50 μg). Treated cells were labeled with CD11b, BODIPY (493/503), CD36, and CD11c and processed through flow cytometry. (A) Representative dot plot of CD36^+^ subsets vs*.* CD11c^+^ subsets. (B) CD36 surface expression shown as mean fluorescence intensity (MFI). (C) BODIPY (493/503) expression shown as mean fluorescence intensity (MFI) with representative histogram. For stimulation studies, cells were pre-treated with CD36 inhibitor SSO (250 μM), or PPARγ agonist rosiglitazone (1 μM), for 60 min and then stimulated with 50 μg HKMT. (D) Representative dot plot of CD36^+^ subsets vs*.* CD11c^+^ subsets. (E) CD11b^+^CD11c^+^CD36^+^ surface expression (%). (F) Representative multiplex western blot for PLIN2 expression. (G) Protein expression of PLIN2 corrected over β-actin. (H) Gene expression of PLIN2 determined by RT-qPCR. (I) BODIPY493/503 expression shown as mean fluorescence intensity (MFI) with representative histogram. (J) Gene expression of CD36 determined by RT-qPCR. (K) Gene expression of CCL2 determined by RT-qPCR. (L) Analysis of secreted inflammatory mediator CCL2 by ELISA. (M) NF-κB reporter monocytic cells SEAP reporter activity. Data are presented as mean ± SEM values (*n* = 3–4, biological replicates) and compared between groups using one-way ANOVA with Tukey’s multiple comparisons test. ∗*p* < 0.05, ∗∗*p* < 0.01, ∗∗∗*p* < 0.001, ∗∗∗∗*p* < 0.0001.

The induction of CD36 expression has been highlighted as the main driving factor for MTB-induced macrophage foaming. Numerous studies consistently demonstrated a substantial decrease in the capacity of foam cells to engulf and eradicate bacteria.^19^^,^^20^ However, therapeutic strategies targeting CD36 remain unsuccessful. In our study, an increase in the percentage of CD11b^+^CD11c^+^CD36^+^ cells was found to be HKMT-dose dependent (Figures 2A and 2B). This upregulation accompanied increased lipid content within treated cells as observed by BODIPY (493/503) flow cytometric analysis (Figure 2C). These observations fall in line with previous reports. In an attempt to further define the importance of CD36 in MTB infection, we conducted a loss/gain of CD36 function analysis by treating cells with either sulfo-*N*-succinimidyl oleate (SSO), a CD36 inhibitor, or rosiglitazone (ROSI), an agonist of the master regulator peroxisome proliferator activated receptor gamma (PPARγ), which therein upregulates CD36. SSO-mediated blockade of CD36 activity did not significantly alter the percentage of CD11b^+^CD11c^+^CD36^+^ cells (Figures 2D and 2E). However, its inhibition significantly reduced the level of intracellular lipid content as observed by PLIN2 protein and gene expression (Figures 2F–2H) and BODIPY (493/503) flow cytometric analysis (Figure 2I). Notably, gene expression analysis revealed a dramatic upregulation of CD36 in SSO-HKMT treated cells (Figure 2J), suggesting that the effect of HKMT on CD36 expression is indirect. These data also suggest that cell foaming is vital during MTB infection. Furthermore, inhibition of CD36 receptor activity severely augmented the inflammatory response induced by HKMT; this was reflected in the significant increase of CCL2 gene and protein expression as well as the enhancement of NF-κB/AP-1 activity. In contrast, no trends in inflammatory marker reduction were observed in ROSI-treated cells (Figures 2K–2M). This observation was also noted in additional inflammatory markers, including TLR2, HIF-1α, IL-1β, and TNF-α, as detailed in the Figures S1A–S1D. It is crucial to note that not all inflammatory markers showed increased levels upon CD36 inhibition as seen in the expression of IL-10 and IL-6 (Figures S1E and S1F). Also, despite the significant gene upregulation in the macrophage inflammatory marker CD11c and the macrophage adhesion molecule CD11b following CD36 inhibition by SSO, HKMT stimulation did not produce any additional synergistic effects (Figures S1G and S1H). We hypothesize that the observed effects are attributable to an indirect inflammatory trigger, likely related to cellular foaming. Collectively, our findings suggest that upregulation of CD36 in MTB infection is not solely regulated at the receptor level. Rather, it involves mechanisms at the transcriptional level. These insights highlight the need for further exploration of the molecular pathways involved in CD36 regulation.

### HKMT modulates CD36 expression through PPARδ activation

**Figure 3:**
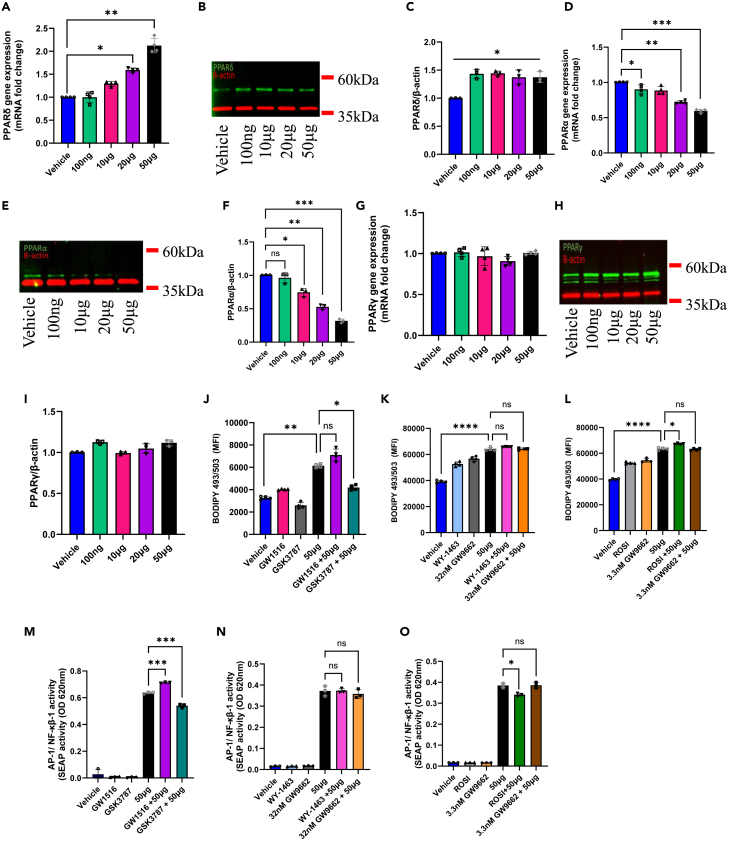
HKMT modulates CD36 expression through PPARδ activation THP-1 cells were treated with several doses of HKMT (0, 100 ng, 10 μg, 20 μg, and 50 μg). Cells were harvested for total mRNA or total protein extraction as described in STAR Methods. (A) Gene expression of PPARδ determined by RT-qPCR. (B) Representative multiplex western blot for PPARδ expression. (C) Protein expression of PPARδ corrected over β-actin. (D) Gene expression of PPARα determined by RT-qPCR. (E) Representative multiplex western blot for PPARα expression. (F) Protein expression of PPARα corrected over β-actin. (G) Gene expression of PPARγ determined by RT-qPCR. (H) Representative multiplex western blot for PPARγ expression. (I) Protein expression of PPARγ corrected over β-actin. For stimulation studies, cells were pre-treated with one of the following: PPARγ agonist rosiglitazone (1 μM), PPARγ antagonist GW9662 (3.3 nM), PPARα agonist WY-14643 (630 nM), PPARα antagonist GW9662 (32 nM), PPARδ agonist GW1516 (1 nM), PPARδ antagonist GSK3787 (1 μM), or suitable vehicle control (0.01% DMSO). Cells were then stimulated with 50 μg of HKMT overnight. (J–L) BODIPY493/503 expression shown as mean fluorescence intensity (MFI). (M–O) NF-κB reporter monocytic cells SEAP reporter activity. Data are presented as mean ± SEM values (*n* = 3–4, biological replicates) and compared between groups using one-way ANOVA with Tukey’s multiple comparisons test. ∗*p* < 0.05, ∗∗*p* < 0.01, ∗∗∗*p* < 0.001, ∗∗∗∗*p* < 0.0001.

PPARs, comprising PPARα, PPARγ, and PPARδ subtypes, serve as ligand-activated master regulators. While previous studies demonstrate the modulation of PPARγ in MTB infection,^21^ the precise mechanisms involving PPARγ and other PPAR family members remain incompletely understood. To investigate the role of PPARs in MTB-induced cell foaming and inflammation, we exposed THP-1 cells to increasing doses of HKMT. Intriguingly, among the three PPAR members, only PPARδ and PPARα exhibited substantial modulation at both gene and protein expression levels upon HKMT stimulation (Figures 3A–3F). In contrast, PPARγ gene expression remained unaffected, with a slight, non-significant increase at protein level (Figures 3G–3I). To further characterize the contribution of PPARs in mitigating lipid accumulation and inflammation, we utilized pharmaceutical agonists/antagonists to modulate PPARs expression in the presence or absence of HKMT stimulation. Notably, PPARδ exerted a minor impact on cell foaming, as its agonist GW1516 showed non-significant trends in increasing lipid content, while its antagonist GSK3787 significantly reduced it (Figure 3J). Although a shift in PPARα expression was observed in response to HKMT in a dose-dependent manner (Figures 3D–3F), no significant effect was observed when employing its agonist WY-1463 or antagonist GW9662 to modulate cell foaming (Figure 3K). Consistent with our prior findings, the activation of PPARγ by its agonist ROSI, in the presence of HKMT stimulation, resulted in substantial cell foaming. However, pre-treating the cells with its antagonist GW9662 (3.3 nM) did not demonstrate a significant preventive effect (Figure 3L). Similarly, modulating PPARδ showed a significant impact on NF-κB/AP-1 activity (Figure 3M), while no notable effect was observed in PPARα modulation (Figure 3N). Interestingly, despite inducing foaming, the activation of PPARγ significantly reduced NF-κB/AP-1 activity. This observation supports the hypothesis that cell foaming serves as a protective mechanism against MTB infection. However, similar to our previous findings, the PPARγ antagonist exhibited no prophylactic effect against HKMT-induced inflammation (Figure 3O). These findings suggest that HKMT-induced cell foaming is reliant on PPARδ activation, whereas the effect on PPARα expression may be indirectly driven by the consequences of lipid accumulation rather than directly influenced by HKMT stimulation.

### PPARδ inhibition and deficiency abrogate HKMT-induced cell foaming and inflammation

**Figure 4:**
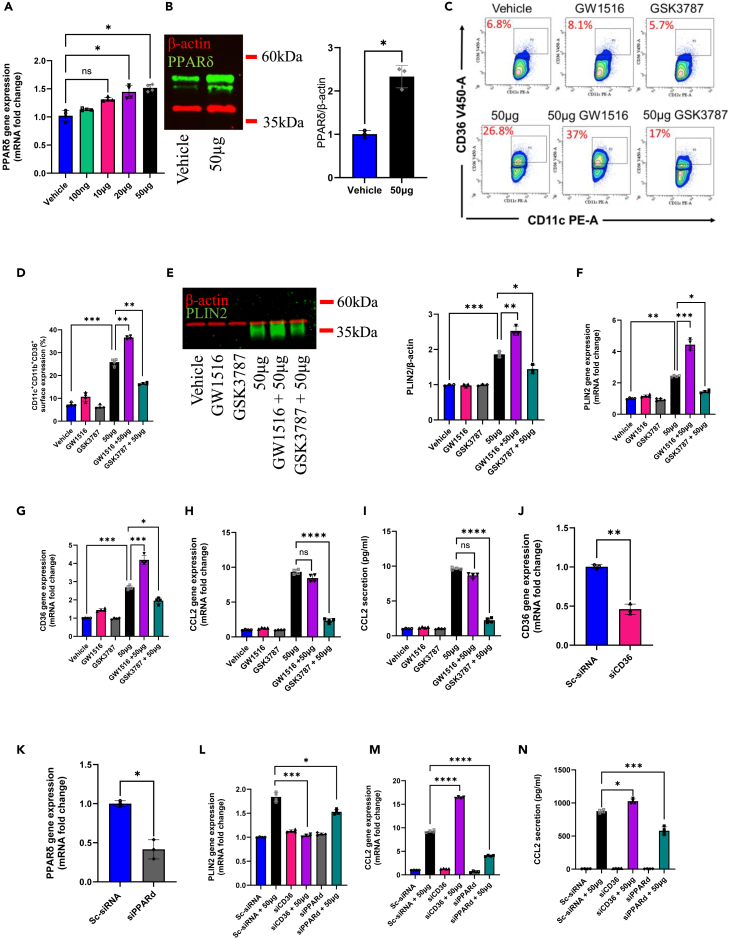
PPARδ inhibition and deficiency abrogate HKMT-induced cell foaming and inflammation Freshly isolated PBMCs from healthy donors were treated with several doses of HKMT (0, 100 ng, 10 μg, 20 μg, and 50 μg). Cells were harvested for total mRNA or total protein extraction as described in STAR Methods. (A) Gene expression of PPARδ was determined using RT-qPCR. (B) Protein expression of PPARδ corrected over β-actin. For a loss/gain of function approach, cells were pre-treated with one of the following: PPARδ agonist GW1516 (1 nM), PPARδ antagonist GSK3787 (1 μM), or suitable vehicle control (0.01% DMSO). Cells were then stimulated with 50 μg of HKMT overnight. (C) Representative dot plot of CD36^+^ subsets vs*.* CD11c^+^ subsets. (D) CD11b^+^CD11c^+^CD36^+^ surface expression (%). (E) Representative multiplex western blot for PLIN2 expression with protein expression of PLIN2 corrected over β-actin. (F) Gene expression of PLIN2 determined by RT-qPCR. (G) Gene expression of CD36 determined by RT-qPCR. (H) Gene expression of CCL2 determined by RT-qPCR. (I) Analysis of secreted inflammatory mediator CCL2 by ELISA. For CD36 and PPARδ genetic silencing studies, transformed THP-1 were transfected with 20 nM each of gene-specific and control (scrambled) siRNAs for 30 h followed by overnight stimulation with 50 μg HKMT. (J) Gene expression of CD36 determined by RT-qPCR. (K) Gene expression of PPARδ determined by RT-qPCR. (L) Gene expression of PLIN2 determined by RT-qPCR. (M) Gene expression of CCL2 determined by RT-qPCR. (N) Analysis of secreted inflammatory mediator CCL2 by ELISA. Data are presented as mean ± SEM values (*n* = 3–4, biological replicates). One-way ANOVA with Tukey’s multiple comparisons test was used to compare between more than two groups, while Student’s t test was used to compare the means between two groups. ∗*p* < 0.05, ∗∗*p* < 0.01, ∗∗∗*p* < 0.001, ∗∗∗∗*p* < 0.0001.

During the course of MTB infection, CD36 expression has been identified as a crucial factor involved in lipid uptake, immunological recognition, and inflammation initiation. Extensive research has focused on the relationship between CD36 and PPARs activation, as CD36 transcription is known to be influenced by PPAR-response elements (PPREs) present in its promoter. However, less is known regarding the interplay between PPARδ and CD36 expression. To explore this dynamic interaction, we identified the impact of HKMT on monocytic cells isolated from PBMCs taken from a healthy donor. HKMT stimulation exerted a dose-dependent induction on PPARδ gene expression (Figure 4A) with a significant upregulation seen in its protein levels when comparing the non-treated healthy cells “vehicle” to those treated with 50 μg HKMT (Figure 4B). Furthermore, we employed a loss/gain of function approach using the PPARδ agonist GW1516 and PPARδ antagonist GSK3787 using our THP-1 model. Remarkably, the activation of PPARδ resulted in a significant increase in the percentage of CD11b^+^CD11c^+^CD36^+^ cells, accompanied by a notable elevation in lipid accumulation, as indicated by PLIN2 protein and gene expression. Conversely, inhibition of PPARδ led to a significant reduction in the percentage of CD11b^+^CD11c^+^CD36^+^ cells, with an observed significant decrease in PLIN2 expression (Figures 4C–4F). Notably, treatment with PPARδ antagonist GSK3787 demonstrated a significant decrease in CD36 gene expression (Figure 4G), implying potential regulatory crosstalk between PPARδ and CD36 at the transcriptional level. Moreover, inhibition of PPARδ significantly attenuated inflammatory polarization, evident from reduced expression of CCL2 (Figure 4H) and the secretion of its protein following GSK3787 treatment (Figure 4I). Collectively, these findings suggest that, like PPARγ, PPARδ exhibits a modulatory effect on CD36 expression and function. Notably, our results also indicate that the induction of CD36 and associated cell foaming serve as a protective mechanism, as evidenced by trends toward reduced inflammation upon CD36 upregulation with ROSI treatment, while inhibition of CD36 using SSO resulted in heightened inflammatory responses (Figures 2F–2M). Furthermore, it becomes apparent that the upregulation of PPARδ is likely responsible for MTB-induced inflammatory and cell foaming response.

To further delineate the individual roles of CD36 and PPARδ during MTB infection, we employed siRNA gene silencing to target these markers, followed by HKMT stimulation. Cells transfected with siRNA targeting CD36 or PPARδ exhibited a significant reduction (≥50%) in CD36 or PPARδ gene expression compared with cells transfected with scrambled siRNA (Figures 4J and 4K, respectively). Targeting CD36 expression resulted in a significant decrease in PLIN2 gene expression, while PPARδ deficiency showed significant reduction at a lower impact on PLIN2, consistent with our previous findings (Figure 4L). Intriguingly, the absence of CD36 led to a significant upregulation of both CCL2 gene and protein secretion, whereas cells deficient in PPARδ exhibited a significant reduction in these markers (Figures 4M and 4N). These results emphasize the relationship between CD36 and PPARδ in MTB infection, highlighting the modulatory roles of PPARδ in lipid accumulation and inflammation, and the protective nature of CD36 induction during infection.

### PPARδ gene expression in monocytes from patients with diabetes correlates with worsened glucose homeostasis and pro-inflammatory cytokine expression

**Figure 5:**
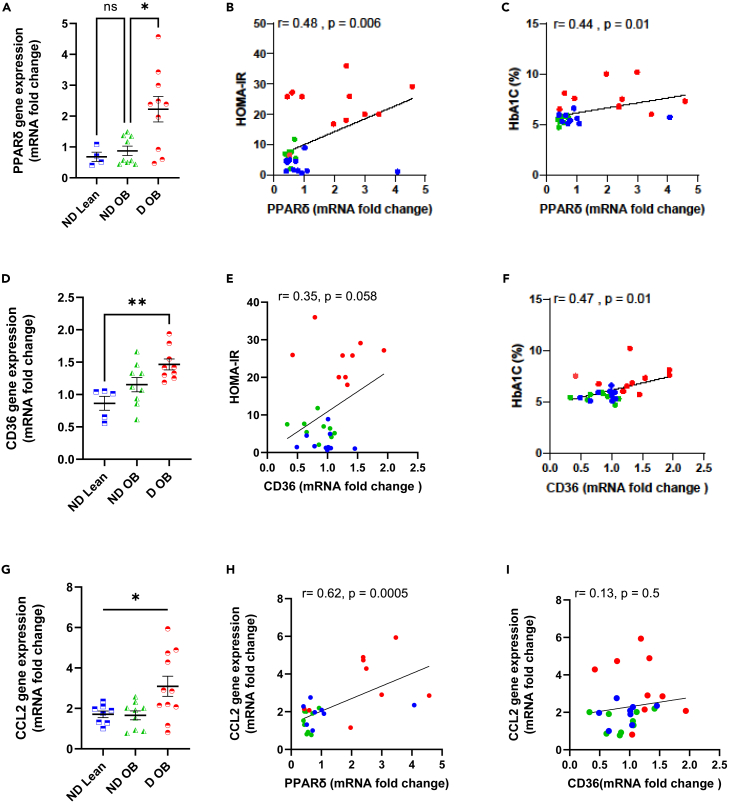
PPARδ gene expression in diabetic patients’ monocytes correlates with worsened glucose homeostasis and pro-inflammatory cytokine expression Monocytic cells were isolated from human blood samples obtained from ND lean (*n* = 10), ND OB (*n* = 11), and obese (*n* = 10) individuals. (A) Gene expression of PPARδ determined by RT-qPCR. (B) Correlation between HOMA-IR and PPARδ mRNA expression in PBMCs of all participants. (C) Correlation between HbA1C (%) and PPARδ mRNA expression in PBMCs of all participants. (D) Gene expression of CD36 determined by RT-qPCR. (E) Correlation between HOMA-IR and CD36 mRNA expression in PBMCs of all participants. (F) Correlation between HbA1C (%) and CD36 mRNA expression in PBMCs of all participants. (G) Gene expression of CCL2 determined by RT-qPCR. (H) Correlation between CCL2 and PPARδ mRNA expression in PBMCs of all participants. (I) Correlation between CCL2 and CD36 mRNA expression in PBMCs of all participants. Data are presented as mean ± SD and compared between groups using one-way ANOVA with Tukey’s multiple comparisons test. Spearman correlation was conducted for non-parametric correlation analysis, while Pearson *r* correlation was conducted for parametric correlation analysis. ∗*p* < 0.05, ∗∗*p* < 0.01, ∗∗∗*p* < 0.001, ∗∗∗∗*p* < 0.0001.

Diabetes significantly increases the likelihood of developing TB, as individuals with diabetes are more prone to experiencing severe manifestations of the disease and facing unfavorable treatment outcomes when compared to those without diabetes. Thus, our objective was to investigate whether the onset of obesity and diabetes affects PPARδ expression in monocytic cells. We compared the expression of PPARδ in lean patients without diabetes (non-diabetes: ND Lean), obese patients without diabetes (ND OB), and obese patients with diabetes (D OB). Remarkably, monocytic cells isolated from D OB patients exhibited significantly higher gene expression of PPARδ compared with ND lean and ND OB controls, while no significant difference was observed between lean and obese non-diabetic controls (Figure 5A). Correlation analysis revealed a significant association between PPARδ expression and both calculating HOMA-IR and HbA1C levels (Figures 5B and 5C, respectively). Notably, when we examined CD36 expression, we observed upregulation between the ND Lean and ND OB groups, as well as an upregulation between the ND OB group and the D OB group. Regardless, the only statistically significant difference was observed between ND Lean and D OB patients (Figure 5D). Furthermore, correlation analysis showed trends of association between HOMA-IR and CD36 expression, while a significant association was found between HbA1C levels and CD36 (Figures 5E and 5F). To gain further insights into the impact of PPARδ and CD36 gene expression on triggering inflammatory signals, we conducted gene expression analysis for the pro-inflammatory marker CCL2. As expected, the gene expression level of CCL2 was significantly higher in D OB patients (Figure 5G). Importantly, only PPARδ gene expression exhibited a significant positive association with elevated CCL2 levels (Figures 5H and 5I).

These findings collectively underscore the existence of an association between PPARδ expression in monocytic cells and the development of diabetes in individuals affected by obesity. The observed subclinical upregulation of PPARδ implies the potential priming of monocytes, which may be further augmented in the presence of PPAR ligands, such as those encountered during MTB infection. This provides a plausible rationale for the increased susceptibility of patients with diabetes to TB following MTB infection. Moreover, these results reinforce the notion that the involvement of CD36 in obesity and diabetes may be primarily attributed to its protective role.

### Validating the role of PPARδ and CD36 in HKMT-induced inflammation using an *ex vivo* model

**Figure 6:**
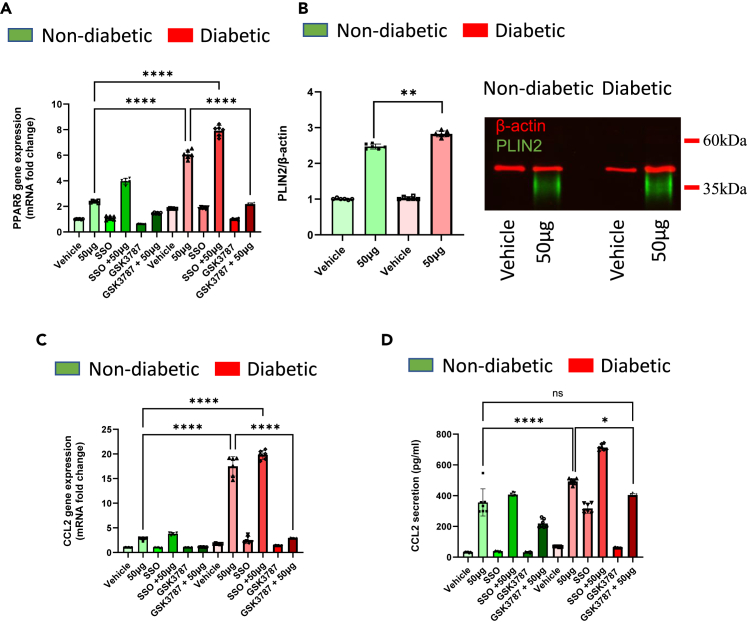
Validating the role of PPARδ and CD36 in HKMT-induced inflammation using an *ex vivo* model Isolated monocytic cells were cultured as previously described and pre-treated with one of the following: CD36 inhibitor SSO (250 μM), PPARδ agonist GW1516 (1 nM), PPARδ antagonist GSK3787 (1 μM), or suitable vehicle control (0.01% DMSO). (A) Gene expression of PPARδ determined by RT-qPCR. (B) Protein expression of PLIN2 with representative multiplex immunoblot. (C) Gene expression of CCL2 determined by RT-qPCR. (D) Analysis of secreted inflammatory mediator CCL2 by ELISA. Data are presented as mean ± SEM values (*n* = 6–7, biological replicates) and compared between groups using one-way ANOVA with Tukey’s multiple comparisons test. ∗*p* < 0.05, ∗∗*p* < 0.01, ∗∗∗*p* < 0.001, ∗∗∗∗*p* < 0.0001.

To investigate the priming effect of PPARδ in patients with diabetes and assess the involvement of CD36 in MTB complications, an *ex vivo* analysis was performed using monocytic cells isolated from both healthy individuals without diabetes and from patients with diabetes. The cultured cells were pretreated with either SSO to inhibit CD36 activity or GSK3787 to inhibit PPARδ. Our findings corroborated previous observations, revealing higher PPARδ gene expression in patients with diabetes, which was further amplified upon HKMT stimulation. Remarkably, the inhibition of PPARδ using GSK3787 significantly reduced its gene expression compared with HKMT stimulation alone in samples from both patients with and without diabetes. Conversely, SSO-mediated inhibition led to a striking elevation in PPARδ gene expression in both groups (Figure 6A). The impact of PPARδ upregulation was further manifested by a significant increase in cell foaming, as evidenced by elevated PLIN2 protein expression in patients with diabetes compared to those without (Figure 6B). Additionally, the upregulation of PPARδ was found to mitigate the HKMT-induced inflammatory response, as indicated by reduced CCL2 gene expression and secretion (Figures 6C and 6D). These findings provide further support for the pivotal role of PPARδ in mediating MTB-induced inflammation, particularly in those with diabetes. Moreover, they underscore the limited involvement of CD36 in this context, suggesting that its role may be primarily associated with a protective response rather than exacerbating inflammation.

## Discussion

TB constitutes a critical, worldwide burden with increasing mortality in patients with diabetes.^22^ The causative agent, *M. tuberculosis*, establishes chronicity via invasion of, replication, and survival within macrophages. This is elaborated through an arsenal of immune evasion and persistence strategies, including inhibition of phagolysosome maturation.^23^ A central feature of MTB infection is the formation of a specific phenotype of macrophages known as “foam cells”.^19^ These are typically associated with accumulation and consumption of various lipid droplets, principally triglycerides, within organized cellular foci known as granulomata.^22^^,^^24^ Several receptors have been reported to contribute to foam cell formation, principally CD36.^25^^,^^26^ Importantly, CD36 is part of a tightly regulated signaling axis involving a family of transcription factors (PPARs, in particular, the PPARγ isoform) that play central roles in macrophage inflammatory responses, cholesterol metabolism, and mitochondrial physiology.^27^^,^^28^^,^^29^ In recent years, the activation of the PPARγ-CD36 axis has increasingly been suggested as a target for therapeutic intervention in the context of various metabolic disorders.^30^ Reports have shown that CD36-dependent accumulation of free fatty acids correlates with a pathophysiological grade of inflammation that perturbs insulin regulation, thereby resulting in reduced insulin sensitivity. Here we show HKMT induced a similar effect in augmentation of lipid accumulation and inflammatory markers compared to those reported with live *M. tuberculosis*.^24^ This observation was shown to be associated with an increased level of CD36 expression, in line with previous reports.^31^ Intriguingly, under the inhibition of CD36 activity by its pharmacological inhibitor SSO, even though we observed significant reduction in intracellular lipid accumulation, an augmented increase in inflammatory response was observed. Further investigation with SSO-mediated pharmacological inhibition of CD36 activity correlated with increased CD36 gene expression. While this phenomenon could potentially stem from a compensatory mechanism triggered by the inhibition of CD36 by SSO, the observed upregulation of CD36 in the presence of HKMT, despite SSO’s functional blockade of the receptor, is consistent with the notion that HKMT exerts its influence on CD36 expression via pathways upstream of the receptor, likely through transcriptional regulatory mechanisms. This hypothesis finds support in numerous studies demonstrating that diverse stimuli can modulate CD36 gene expression independently of receptor activity, often mediated by transcription factors such as PPARs.^32^^,^^33^^,^^34^

Following on from these observations, we investigated the expression profiles of PPARs in cells treated with various pharmacological agonists and antagonists. In contrast to earlier reports supporting a role for PPARγ in regulating CD36 expression during tuberculosis,^35^^,^^36^ our results could not demonstrate a significant involvement of PPARγ in HKMT-mediated modulation of CD36 expression. Rather, we found a significant effect for PPARδ. These discrepancies may be explained in part by the cell model and bacterial strain, in that BCG (rather than HKMT) and mouse peritoneal macrophages (rather than THP-1 cells) were used in other reports.^35^^,^^36^ Regardless, in HKMT-exposed THP-1 cells, treatment with the PPARδ antagonist GSK3787 or gene silencing through siRNA technology correlated with a significant reduction in CD36 gene expression; this was paralleled by significantly reduced lipid accumulation as well as inflammation. This observation highlights the relations between other PPAR family members and CD36. Indeed, it is well documented that at the transcriptional level, PPARs (more specifically, PPAR-γ) and RXRα heterodimerize and bind to the promoter of the CD36 gene and positively control its transcription.^37^ However, even though other family members’ interaction is less investigated, few studies have shown that CD36 can modulate other members. In a work presented by Lujin Wu et al., it was demonstrated that the PPARα-CD36 pathway was found to be a major modulator of cardiac fatty acid metabolism, influencing fatty acid uptake and oxidation in a diabetic cardiomyopathy mouse model. This interaction has also been implicated in regulating lipid homeostasis and oxidative stress in other tissues, including the liver.^38^^,^^39^ Additionally, CD36 has been found to exhibit crosstalk with PPARδ in macrophages, impacting foam cell formation and inflammation in atherosclerosis and in induced inflammation from *M. leprae* infection models.^40^^,^^41^^,^^42^

To further understand the role of PPARδ in the pathophysiology of TB in the setting of diabetes, we first examined its expression in monocytic cells isolated from lean controls without diabetes, as well as OB individuals with or without diabetes. Importantly, we showed elevated gene expression of PPARδ, CD36, and CCL2 in monocytic cells from OB individuals with diabetes compared with those without. Intriguingly, correlation analysis showed a significant association between PPARδ, but not CD36, and CCL2, therein implicating subclinical inflammatory potentiation of monocytic cells in patients with diabetes. In parallel, we observed a strong association between PPARδ and markers of worsened glucose homeostasis seen in the elevation of HOMA-IR and HbA1C levels in individuals with diabetes compared to those without.

We then isolated PBMCs from individuals with or without diabetes and pretreated them with either SSO to inhibit CD36 or GSK3787 to inhibit PPARδ before exposing them to HKMT. We observed a striking and significant reduction in PPARδ gene expression in cells treated with both GSK3787 and HKMT compared with cells exposed only to HKMT. This was evident in both patient groups. Conversely, treatment with SSO followed by HKMT stimulation correlated with increased PPARδ gene expression. Importantly, we found that cells pretreated with GSK3787 prior to HKMT exposure showed a stronger and more significant reduction in inflammatory marker expression compared with cells exposed to HKMT only. These observations hint at a complex interplay between PPARδ and CD36 in regulating the foaming and inflammatory response of macrophages during tuberculosis, particularly in diabetic patients. Moreover, the data suggest a protective, rather than pathological, role for CD36 in this specific context.

More work is needed to definitively identify the mycobacterial component that elicits the activation of the CD36-PPARδ signaling axis, especially in the context of diabetes. A major virulence factor synthesized in the mycobacterial cell wall is lipoarabinomannan (LAM); this consists of a mannosyl-phosphatidyl-*myo*-inositol plasma membrane anchor (MPI) connected to a mannan core, followed by an arabinan domain.^43^^,^^44^ Depending on the pathogenic species from which it is isolated, LAM displays various structural motifs at its nonreducing ends. In *M. tuberculosis*, LAM is mannosylated (hence called mannosylated LAM, or ManLAM) and features α(1–2)-linked oligomannosides at its nonreducing ends.^45^ Although TLRs (particularly TLR2 and TLR4) are known to mediate *M. tuberculosis*-dependent inflammatory activation of macrophages,^46^^,^^47^ it appears ManLAM exerts anti-inflammatory effects independently of TLR2. Moreover, it has been postulated that steric hindrance prevents physical binding of ManLAM to TLR2 and that C-type lectins, in particular DC-SIGN, recognize the oligomannoside-binding cap of ManLAM; the engagement of DC-SIGN with ManLAM during phagocytic uptake has been reported to contribute to cessation of phagolysosome formation and suppression of inflammation.^48^^,^^49^ It would thus be intriguing to definitively demonstrate signaling crosstalk between ManLAM, DC-SIGN, CD36, and PPARδ in perpetuating and/or suppressing *M. tuberculosis*-mediated inflammation and macrophage foaming in the diabetic setting.

In conclusion, our findings illuminate a multifaceted role for the CD36-PPARδ signaling axis in the context of TB, especially in diabetic conditions. The revelation that HKMT activates CD36 and its pivotal role in lipid accumulation underscores the intricacies of the host’s response to the bacterium. Surprisingly, while CD36 inhibition diminished lipid levels, it failed to alleviate the inflammatory response, suggesting a decoupling of lipid accumulation from inflammation. The upregulation of PPARδ, especially under CD36 inhibition, highlights its potential role in this complex scenario. Our exploration into the CD36-PPARδ axis led us to postulate that while CD36 might be geared toward protective mechanisms, its interaction with PPARδ could inadvertently exacerbate inflammation. This is especially relevant given our findings in diabetic PBMCs, which exhibited heightened PPARδ expression and consequent elevated inflammation. The therapeutic potential of PPARδ antagonists, as demonstrated by the reduction in inflammation upon treatment with GSK3787, offers promising avenues for TB treatment in diabetic patients.

### Limitations of the study

While our study provides valuable insights into the intricate interplay between the CD36-PPARδ signaling axis in tuberculosis (TB), particularly in the context of diabetes, several limitations should be acknowledged. Foremost, the use of heat-killed *Mycobacterium tuberculosis* (HKMT) instead of live *M. tuberculosis* may restrict the direct translation of our findings to *in vivo* settings. Live bacteria present a dynamic and evolving challenge to the host immune system, which may elicit responses distinct from those triggered by heat-killed counterparts. Additionally, our focus on HKMT limits our ability to fully capture the nuanced interactions between host factors and live mycobacterial components, such as LAM, a key virulence factor. LAM’s role in modulating inflammatory responses and macrophage function remains incompletely understood, particularly in diabetic individuals. Furthermore, our study primarily relies on *in vitro* models, namely THP-1 cells and PBMCs, which may not fully recapitulate the complex cellular milieu present *in vivo*. Additionally, while our findings suggest a potential therapeutic role for PPARδ antagonists in mitigating inflammation, further preclinical and clinical investigations are warranted to elucidate the efficacy and safety of such interventions in TB patients, especially those with concomitant diabetes. Despite these limitations, our study underscores the importance of exploring novel therapeutic targets and understanding the intricate host-pathogen interactions in the context of TB and diabetes co-morbidity.

## STAR★Methods

### Key resources table

**Table:** 

REAGENT or RESOURCE	SOURCE	IDENTIFIER
**Deposited data**		

Original WB immunoblots images	Mendeley Data	https://doi.org/10.17632/khhdrs48m2.1

**Antibodies**		

anti-CD11c (S-HCL-3) PE	BD Biosciences	Cat#340713
anti-CD11b (D12)-APC	BD Biosciences	Cat#340936
anti-CD36 V450-A	BD HORIZON	Cat#561535
BD Phosflow™ Alexa Fluor®488 Mouse IgG2a, κ Isotype control	BD Biosciences	Cat#558055; RRID: AB_1645612
BD Pharmingen™ PE Mouse IgG2b, κ Isotype Control	BD Biosciences	Cat#559529; RRID: AB_397261
BD Pharmingen™ PE-Cy™7 Mouse IgG2b, κ Isotype Control	BD Biosciences	Cat#560542; RRID: AB_1727595
BD Phosflow™ Alexa Fluor® 647 Mouse anti-IκBα	BD Biosciences	Cat#560817; RRID: AB_2033974
BODIPY™ 493/503 (4,4-Difluoro-1,3,5,7,8-Pentamethyl-4-Bora-3a,4a-Diaza-s-Indacene)	Life Technologies∖ ThermoFisher	Cat# D3922
Anti-PPAR alpha (phospho S12) antibody	abcam	Cat#ab3484
Anti-PPAR delta antibody	abcam	Cat#ab8937
Anti-PPAR gamma antibody	abcam	Cat#ab209350
Perilipin-2/ADFP Antibody - BSA Free	NOVUS	Cat#NB110-40878
β-Actin (8H10D10) Mouse mAb	Cell Signaling	Cat#3700
Goat Anti-Rabbit IgG H&L (IRDye® 680RD) preadsorbed	abcam	Cat#ab216777
Goat anti-Mouse IgG H&L (IRDye® 800CW) preadsorbed	abcam	Cat#ab216772

**Bacterial and virus strains**		

Heat-killed Mycobacterium tuberculosis (HKMT)	InvivoGen	Cat# tlrl-hkmt-1

**Biological samples**		

Human Peripheral Blood Mononuclear Cell (PBMC)	Dasman Diabetes Institute	N/A

**Chemicals, peptides, and recombinant proteins**		

Sulfo-N-succinimidyl Oleate sodium (SSO)	Sigma-Aldrich	Cat#SML2148; CAS: 135661-44-8
PPARγ agonist Rosiglitazone	Merkmillipore	Cat#557366; CAS: 155141-29-0
PPARγ antagonist GW9662	Sigma-Aldrich	Cat#M6191; CAS: 22978-25-2
PPARα agonist WY-14643	biotechne	Cat#1312; CAS:50892-23-4
PPARα antagonist GW9662	biotechne	Cat#1508; CAS: 22978-25-2
PPARδ agonist GW501516	Sigma-Aldrich	Cat#SML1491
PPARβ/δ Antagonist, GSK3787	Sigma-Aldrich	Cat#516567; CAS:188591-46-0

**Critical commercial assays**		

RNeasy Mini Kit	QIAGEN	Cat#74104
High-capacity cDNA reverse transcription kit	Applied Biosystems	Cat#43-688-14
TaqMan® Gene Expression Master Mix	Applied Biosystems	Cat#43-690-16
Cell Line Nucleofector™ Kit V	Lonza	Cat#11586329
Human CCL2/MCP-1 DuoSet ELISA	biotechne	Cat#DY279

**Experimental models: Cell lines**		

Human monocytic THP-1 cells	ATCC	Cat#TIB-202
THP1-Blue™ NF-κB Cells	InvivoGen	Cat#thp-nfkb

**Oligonucleotides**		

CD36 Human siRNA Oligo Duplex (Locus ID 948)	ORiGENE	Cat#SR319610
PPAR delta (PPARD) Human siRNA Oligo Duplex (Locus ID 5467)	ORiGENE	Cat#SR303654
siRNA Control Trilencer-27 Universal scrambled negative control siRNA duplex - 2 nmol	ORiGENE	Cat#SR30004
Primers for: PLIN2	Thermofisher	Cat#4331182; Assay ID:Hs00605340_m1
Primers for: CCL2	Thermofisher	Cat# 4331182; Assay ID:Hs00234140_m1
Primers for: CD36	Thermofisher	Cat# 4331182; Assay ID:Hs00354519_m1
Primers for: PPAR GAMMA	Thermofisher	Cat#4331182; Assay ID:Hs01115513_m1
Primers for: PPAR DELTA	Thermofisher	Cat# 4331182; Assay ID:Hs04187066_g1
Primers for: PPAR ALPHA	Thermofisher	Cat#4331182; Assay ID: Hs00947536_m1
Primers for: TLR2	Thermofisher	Cat#4331182; Assay ID: Hs00152932_m1
Primers for: HIF-1α	Thermofisher	Cat#4331182; Assay ID:
Primers for: IL-1β	Thermofisher	Cat#4331182; Assay ID: Hs01555410_m1
Primers for: TNF-α	Thermofisher	Cat#4331182; Assay ID: Hs01113624_g1
Primers for: GAPDH	Thermofisher	Cat#4331182; Assay ID:Hs03929097_g1

**Software and algorithms**		

FlowJo	BD Biosciences	https://www.bdbiosciences.com/en-eu/products/software/instrument-software/bd-facsdiva-software
GraphPad Prism software	Dotmatics	https://www.graphpad.com/features
Image Lab Software	BIO-RAD	https://www.bio-rad.com/en-kw/product/image-lab-software?ID=KRE6P5E8Z
GraphPad Prism software	GraphPad	https://www.graphpad.com/

**Other**		

BioRender	BioRender	https://www.biorender.com/
BD FACSCanto™ II Clinical Flow Cytometry System	Biosciences	https://www.bdbiosciences.com/en-eu/products/instruments/flow-cytometers/clinical-cell-analyzers/facscanto
QuantStudio™ 7 Pro Real-Time PCR System, 96-well, 0.2 mL, desktop	ThermoFisher	Cat#A43183; https://www.thermofisher.com/order/catalog/product/A43183
amaxa Nucleofector ® II	Lonza	https://bioscience.lonza.com/nucleofector-technology?_bt=658313049255&_bk=amaxa%20electroporation&_bm=e&_bn=g&_bg=148300625719&gad_source=1&gclid=EAIaIQobChMI_cLh6LyTggMVF4poCR2HUgOEEAAYASAAEgKsi_D_BwE
ChemiDoc MP Imaging System	BIO-RAD	Cat#12003154; https://www.bio-rad.com/en-kw/product/chemidoc-mp-imaging-system?ID=NINJ8ZE8Z#:∼:text=The%20ChemiDoc%20MP%20Imaging%20System%20is%20a%20full%2Dfeature%20instrument,stain%2Dfree%20technology%20imaging%20needs.

### Resource availability

#### Lead contact

Further information and requests for resources and reagents should be directed to and will be fulfilled by the Lead Contact, Fatema Al-Rashed (fatema.alrashed@dasmaninstitute.org).

#### Materials availability

This study did not generate new unique reagents.

#### Data and code availability

• Original western blot images have been deposited at Mendeley and are publicly available as of the date of publication. The DOI is listed in the key resources table.
• This paper does not report any original code.
• Any additional information required to reanalyze the data reported in this paper is available from the lead contact upon request.

### Experimental model and study participant details

#### Cell culture

Human monocytic THP-1 cells were purchased from American Type Culture Collection (ATCC) and grown in RPMI-1640 culture medium (Gibco, Life Technologies, Grand Island, USA) supplemented with 10% fetal bovine serum (Gibco), 2 mM glutamine (Gibco), 1 mM sodium pyruvate, 10 mM HEPES, 100 ug/ml Normocin, 50 U/ml penicillin and 50 μg/ml streptomycin (P/S; (Gibco). They were incubated at 37°C (with humidity) in 5% CO_2_.

#### Cell stimulation

Monocytic cells were plated in 12-well plates (Costar, Corning Incorporated, Corning, NY, USA) at 1×10^6^ cells/well unless indicated otherwise. To induce dose-dependent stimulation, several doses (0, 100 ng, 10 μg, 20 μg and 50 μg) of Heat-killed *Mycobacterium tuberculosis* (HKMT) (Invivogen, San Diago, USA; tlrl-hkmt-1) were used. Cells were harvested for either total mRNA or protein isolation, and the culture media were collected for measuring cytokine secretion. For various stimulation studies, cells were pre-treated for 1 hour with 250 μM of CD36 inhibitor sulfo-*N*-succinimidyl oleate (SSO; Sigma-Aldrich SML2148), PPARγ agonist rosiglitazone (1 μM; Millipore 557366-M), PPARγ antagonist GW9662 (3.3 nM; Sigma-Aldrich M6191 ), PPARα agonist WY-14643 (630 nM), PPARα antagonist GW9662 (32 nM; biotechne TOCRIS 1312 ), PPARδ agonist GW1516 (1 nM ; Sigma-Aldrich SML1491 ), PPARδ antagonist GSK3787 (1 μM; Sigma-Aldrich 516567 ) or suitable vehicle control (0.01% DMSO). Cells were then stimulated with 50 μg of HKMT overnight.

#### Participant details

In this study, a total of 31 participants (12 males / 19 females) of Kuwaiti nationality were recruited from the outpatient clinic with a mean age of 46 ± 9 years. The participants were divided as follows: 10 non-diabetic normal weights with BMI ≤ 25 kg/m^2^, 11 non-diabetic obese controls with BMI ≥ 30 kg/m^2^, and 10 patients with type 2 diabetes (T2DM) with BMI ≥ 30 kg/m^2^. The T2D patients were identified based on their fasting plasma glucose level of 7.0 mmol/L and the use of anti-diabetic medication. Individuals with significant underlying health conditions such as pulmonary, renal, hepatic, cardiovascular, hematologic, or immune diseases, as well as those with type 1 diabetes, pregnancy, or malignancy, were excluded from the study. While all participants were of Kuwaiti nationality, specific data on ancestry, race, and ethnicity were not collected as these were not part of the inclusion and exclusion criteria. The participant groups may have diverse ancestry, race, and ethnicity, reflecting Kuwait's richly diverse population.

The clinical characteristics of the participants can be found in Table S1, while bloodwork markers are summarized in Table S2. Prior to their involvement in the study, all participants were provided with information about the study's purpose and gave written informed consent. The study was conducted in accordance with the ethical guidelines outlined in the Declaration of Helsinki and received approval from both the Kuwait Ministry of Health Ethical Board (Approval ID#: 1806/2021) and the Ethical Review Committee (ERC) of Dasman Diabetes Institute, Kuwait (Approval ID#: RA MoH-2022-002; RA 2010-003). No significant differences were observed between the non-diabetic lean control group and the non-diabetic obese group in terms of HOMA-IR assessments, which were calculated based on fasting glucose and insulin secretion or their HbA1c%. As expected, the T2D patients exhibited higher levels of insulin resistance compared to the non-diabetic participants. This was indicated by their HOMA-IR values (*p* < 0.0001) and elevated levels of HbA1c% (*p* = 0.0004), a common marker used to assess the degree of T2D control.

### Method details

#### Isolation of human peripheral blood mononuclear cells (PBMC) and monocyte purification

Peripheral blood mononuclear cells (PBMCs) were isolated from non-diabetic and diabetic participants in EDTA vacutainer tubes. PBMCs were isolated using the HistoPaque density gradient method and Sepmate Tube (StemCell Technologies, Vancouver, CN, Canada) as previously described.^50^^,^^51^ To generate monocytic cells, PBMCs were plated in 6-well plates unless specified otherwise (Costar) at 3 × 10^6^ cells/well and cultured in RPMI-1640 culture medium (Gibco) supplemented with 2 mM glutamine (Gibco), 1 mM sodium pyruvate, 10 mM HEPES, 100 ug/mL Normocin, 50 U/ml penicillin, and 50 μg/mL streptomycin (P/S; (Gibco). Cells were incubated at 37°C (with humidity) in 5% CO_2_ for 3 h. Non-adhered cells were removed, and monocytes adhered to the plate were washed with serum-free culture medium and later incubated for 24 h in RPMI with 10% fetal bovine serum. For stimulation purposes, medium was replaced with fresh medium prior to inhibition and HKMT stimulation as previously described. Cells were pre-treated with inhibitors followed by stimulation with HKMT as previously described.

#### Flow Cytometry—Staining of cell-surface markers

Monocytic cells were seeded in 24-well plates at 0.5x10^5^ cell/ml. Cells were pre-treated with inhibitors as previously described then subjected to stimulation with HKMT or PBS (vehicle) overnight. Cells were washed in ice-cold PBS then resuspended in FACS staining buffer (BD Biosciences) and blocked with human IgG (Sigma; 20 μg) for 30 minutes on ice. Cells were washed and resuspended in 100 μl of FACS buffer and incubated with anti-CD11c (S-HCL-3) PE (cat # 340713; BD Biosciences ) , anti-CD11b (D12)-APC (cat # 340936; BD Biosciences ) and anti-CD36 V450-A (cat # 561535; BD HORIZON ) or suitable isotype control antibody (Cat # 558055; BD Phosflow™, Cat # 559529; BD Phosflow™ , Cat # 560542; BD Pharmingen™ or Cat # 560817; BD Phosflow™) on ice for 30 minutes. BODIPY (495/503) stain was used to quantify lipid levels. BODIPY (495/503) was purchased from Life Technologies (D3922). Cells were washed three times in FACS buffer and resuspended in 2% paraformaldehyde. Cells were centrifuged and resuspended in FACS buffer for FACS analysis (FACSCanto II; BD Bioscience, San Jose, USA). FACS data analysis was performed using BD FACSDivaTM Software 8 (BD Biosciences, San Jose, USA) or FlowJo. Unstained cells were used to set the quadrant of the negative vs positive gates. The stain index (SI) was calculated as the difference between the mean fluorescence intensity of the positive and negative populations, divided by two times the standard deviation of the negative populations ‘unstained cells. Gating strategy is summarized in Figure S2.

#### Quantitative Real-Time Polymerase Chain Reaction (qRT-PCR)

Total RNA was extracted using a RNeasy Mini Kit (Qiagen, Valencia, CA, USA) as per the manufacturer’s instructions and as previously described.^40^ cDNA was synthesized from 1 μg total RNA using the high-capacity cDNA reverse transcription kit (Applied Biosystems, Foster city, CA, USA). qRT-PCR was performed on a QuantStudio™ 7 Pro Real-Time PCR System (Applied Biosystems) using a TaqMan® Gene Expression Master Mix (Applied Bio-systems). Each reaction contained 50 ng cDNA that was amplified with inventoried TaqMan Gene Expression Assay products (PLIN2: Assay ID: Hs00605340_m1; CCL2: Assay ID: Hs00234140_m; CD36: Assay ID: Hs00354519_m1; GAPDH: Assay ID: Hs03929097_g1; PPARA: Assay ID: Hs00947536_m1; PPARG: Assay ID: Hs01115513_m; PPARD: Assay ID: Hs04187066_g1). The threshold cycle (Ct) values were normalized to the housekeeping gene GAPDH, and the amounts of target mRNA relative to control were calculated using the ΔΔ^*Ct*^-method. Relative mRNA expression was expressed as fold expression over the average of control gene expression. The expression level in control treatment was set at 1. Values are presented as mean ± SEM. Results were analyzed statistically; *p* < 0.05 was considered significant.

#### Small-Interfering RNA (siRNA) transfection

Monocytes were washed and resuspended in 100 μl of nucleofector solution provided with the Amaxa Noclecfector Kit V and transfected separately with 30 nM siRNA-CD36 (Cat # CAT#: SR319610), siRNA-PPARδ (Cat # SR303654), or scramble (control) siRNA (OriGene Technologies, Inc. MD, USA, USA), and pmaxGFP (0.5 μg; Amaxa Noclecfector Kit V for THP-1, Lonza). All transfection experiments were performed with Amaxa Cell Line Nucleofector Kit V for monocytic cells (Lonza, Germany) by using Amaxa Electroporation System (Amaxa Inc, Germany) according to the manufacturer's protocol. After 36 hours of transfection, cells were considered ready and were cultured and treated as described previously. CD36 and PPARδ gene knock down level was assessed by qRT-PCR.

#### Measurement of AP-1/NF-κB activity

AP-1/NF-kB reporter monocytes (THP-1 XBlue; InvivoGen, San Diego, CA) are stably transfected with a reporter construct, expressing a secreted embryonic alkaline phosphatase (SEAP) gene under the control of a promoter inducible by the transcription factors AP-1 and NF-κB. Upon stimulation, NF-κB is activated and subsequently the secretion of SEAP is stimulated. Cells were pretreated as described previously. Levels of SEAP were detected in the culture media 3 hours after incubating supernatants with Quanti-Blue solution (InvivoGen) at 650 nm by ELISA reader.

#### Multiplex fluorescent western blotting

Isolated PBMCs or harvested cells were incubated for 30 min with lysis buffer (Tris 62.5 mM, pH 7.5, 1% Triton X-100, and 10% glycerol). The lysates were centrifuged at 14,000× *g* for 10 min and the supernatants were collected. Protein concentration in lysates was measured by Quickstart Bradford Dye Reagent, 1x Protein Assay kit (Bio-Rad Laboratories, Inc, CA). Protein samples (50 μg) were mixed with 20 μl sample loading buffer, heated for 5 min at 95°C, and resolved on 12% polyacrylamide gels using SDS-PAGE. Cellular proteins were transferred to Immuno-Blot Nitrocellulose membranes (Bio-Rad Laboratories) by electroblotting. The membranes were then blocked with 5% non-fat milk in PBS for 30-45 min, followed by incubation with primary antibodies against PPARα (MW: 52 kDa; Abcam Cat # ab3484), PPARδ (MW: 60-50 kDa; Abcam Cat # ab8937 ), PPARγ (MW: 57 and 54 kDa; Abcam Cat # ab209350), PLIN2 (MW: 40 kDa, NOVUS Cat # NB110-40878) or β-actin (MW: 42 kDa; Cell signalling technology Cat # 3700T) at a 1:1000 dilution at 4°C overnight. The blots were then washed four times in TBST (PBS + 0.1% Tween-20) followed by second incubation with RDye680 (Abcam Cat #ab216777) - or IRDye800 (Abcam Cat # ab216772) -conjugated secondary antibodies (1:5000) for 1 h at room temperature. Membranes were washed again with TBST three times followed by one time with TBS with no Tween-20. Signals were visualized by Molecular Imager® ChemiDoc™ MP Imaging Systems (Bio-Rad Laboratories) and quantified using Image Lab Software from Bio-Rad Laboratories. For densitometric analysis, signal intensities of target proteins were normalized to the loading control β-actin.

#### Sandwich enzyme-linked immunosorbent assay (ELISA)

Secreted CCL2 protein concentrations were quantified in the supernatants of PBMC-derived monocytes and THP-1 treated cell cultures using sandwich ELISA in accordance with the manufacturer’s instructions (R&D systems, Minneapolis, MN, USA , Cat # DY279) and as previously published.^40^

### Quantification and statistical analysis

Statistical analysis was performed using GraphPad Prism software (La Jolla, CA, USA). Data are shown as the mean ± the standard error of the mean (SEM), unless otherwise indicated. Parametric data were analyzed by one-way ANOVA followed by Tukey’s post hoc multiple comparisons test. For non-parametric data, the exact Kruskal-Walli’s test was used. The correlation between CD36 and PPARδ gene expression and glucose homeostasis parameters (HOMA-IR and HbA1C) was evaluated with Spearman’s correlation coefficient test, while the correlation between the parametric association between PPARδ and CD36 gene expression and CCL2 gene expression was investigated by Pearson correlation coefficient analysis. For all analyses, data from a minimum of three replicates were used for statistical calculation. A *p* value of 0.05 was considered statistically significant. ns = non-significant, ∗ *p* < 0.05, ∗∗ *p* < 0.01, ∗∗∗ *p* < 0.001, and ∗∗∗∗ *p* < 0.0001.

## References

[1] Chandra P., Grigsby S.J., Philips J.A. (2022). Immune evasion and provocation by Mycobacterium tuberculosis. Nat. Rev. Microbiol..

[2] Nowiński A., Wesołowski S., Korzeniewska-Koseła M. (2023). The impact of comorbidities on tuberculosis treatment outcomes in Poland: a national cohort study. Front. Public Health.

[3] Adane H.T., Howe R.C., Wassie L., Magee M.J. (2023). Diabetes mellitus is associated with an increased risk of unsuccessful treatment outcomes among drug-susceptible tuberculosis patients in Ethiopia: A prospective health facility-based study. J. Clin. Tuberc. Other Mycobact. Dis..

[4] Baker M.A., Harries A.D., Jeon C.Y., Hart J.E., Kapur A., Lönnroth K., Ottmani S.E., Goonesekera S.D., Murray M.B. (2011). The impact of diabetes on tuberculosis treatment outcomes: a systematic review. BMC Med..

[5] Kim H., Shin S.J. (2023). Revolutionizing control strategies against Mycobacterium tuberculosis infection through selected targeting of lipid metabolism. Cell. Mol. Life Sci..

[6] Fines D.M., Schichnes D., Knight M., Anaya-Sanchez A., Thuong N., Cox J., Stanley S.A. (2023). Mycobacterial formation of intracellular lipid inclusions is a dynamic process associated with rapid replication. bioRxiv.

[7] Vrieling F., Wilson L., Rensen P.C.N., Walzl G., Ottenhoff T.H.M., Joosten S.A. (2019). Oxidized low-density lipoprotein (oxLDL) supports Mycobacterium tuberculosis survival in macrophages by inducing lysosomal dysfunction. PLoS Pathog..

[8] Korb V.C., Chuturgoon A.A., Moodley D. (2016). Mycobacterium tuberculosis: Manipulator of Protective Immunity. Int. J. Mol. Sci..

[9] Silverstein R.L., Febbraio M. (2009). CD36, a scavenger receptor involved in immunity, metabolism, angiogenesis, and behavior. Sci. Signal..

[10] Philpott D.J., Sorbara M.T., Robertson S.J., Croitoru K., Girardin S.E. (2014). NOD proteins: regulators of inflammation in health and disease. Nat. Rev. Immunol..

[11] Hawkes M., Li X., Crockett M., Diassiti A., Finney C., Min-Oo G., Liles W.C., Liu J., Kain K.C. (2010). CD36 deficiency attenuates experimental mycobacterial infection. BMC Infect. Dis..

[12] Maglione P.J., Chan J. (2009). How B cells shape the immune response against Mycobacterium tuberculosis. Eur. J. Immunol..

[13] Court N., Vasseur V., Vacher R., Frémond C., Shebzukhov Y., Yeremeev V.V., Maillet I., Nedospasov S.A., Gordon S., Fallon P.G. (2010). Partial redundancy of the pattern recognition receptors, scavenger receptors, and C-type lectins for the long-term control of Mycobacterium tuberculosis infection. J. Immunol..

[14] Liu L., Liu J., Niu G., Xu Q., Chen Q. (2015). Mycobacterium tuberculosis 19-kDa lipoprotein induces Toll-like receptor 2-dependent peroxisome proliferator-activated receptor γ expression and promotes inflammatory responses in human macrophages. Mol. Med. Rep..

[15] Mahajan S., Dkhar H.K., Chandra V., Dave S., Nanduri R., Janmeja A.K., Agrewala J.N., Gupta P. (2012). Mycobacterium tuberculosis modulates macrophage lipid-sensing nuclear receptors PPARγ and TR4 for survival. J. Immunol..

[16] Wang Y., He X., Zheng D., He Q., Sun L., Jin J. (2023). Integration of Metabolomics and Transcriptomics Reveals Major Metabolic Pathways and Potential Biomarkers Involved in Pulmonary Tuberculosis and Pulmonary Tuberculosis-Complicated Diabetes. Microbiol. Spectr..

[17] Silva A.R., Gonçalves-de-Albuquerque C.F., Pérez A.R., Carvalho V.d.F. (2019). Immune-endocrine interactions related to a high risk of infections in chronic metabolic diseases: The role of PPAR gamma. Eur. J. Pharmacol..

[18] Shim D., Kim H., Shin S.J. (2020). Mycobacterium tuberculosis Infection-Driven Foamy Macrophages and Their Implications in Tuberculosis Control as Targets for Host-Directed Therapy. Front. Immunol..

[19] Bedard M., van der Niet S., Bernard E.M., Babunovic G., Cheng T.Y., Aylan B., Grootemaat A.E., Raman S., Botella L., Ishikawa E. (2023). A terpene nucleoside from M. tuberculosis induces lysosomal lipid storage in foamy macrophages. J. Clin. Invest..

[20] Peyron P., Vaubourgeix J., Poquet Y., Levillain F., Botanch C., Bardou F., Daffé M., Emile J.F., Marchou B., Cardona P.J. (2008). Foamy macrophages from tuberculous patients' granulomas constitute a nutrient-rich reservoir for M. tuberculosis persistence. PLoS Pathog..

[21] Arnett E., Weaver A.M., Woodyard K.C., Montoya M.J., Li M., Hoang K.V., Hayhurst A., Azad A.K., Schlesinger L.S. (2018). PPARγ is critical for Mycobacterium tuberculosis induction of Mcl-1 and limitation of human macrophage apoptosis. PLoS Pathog..

[22] Agarwal P., Gordon S., Martinez F.O. (2021). Foam Cell Macrophages in Tuberculosis. Front. Immunol..

[23] Zhai W., Wu F., Zhang Y., Fu Y., Liu Z. (2019). The Immune Escape Mechanisms of *Mycobacterium Tuberculosis*. Int. J. Mol. Sci..

[24] Agarwal P., Combes T.W., Shojaee-Moradie F., Fielding B., Gordon S., Mizrahi V., Martinez F.O. (2020). Foam Cells Control *Mycobacterium tuberculosis* Infection. Front. Microbiol..

[25] Febbraio M., Hajjar D.P., Silverstein R.L. (2001). CD36: a class B scavenger receptor involved in angiogenesis, atherosclerosis, inflammation, and lipid metabolism. J. Clin. Invest..

[26] Stuart L.M., Deng J., Silver J.M., Takahashi K., Tseng A.A., Hennessy E.J., Ezekowitz R.A.B., Moore K.J. (2005). Response to Staphylococcus aureus requires CD36-mediated phagocytosis triggered by the COOH-terminal cytoplasmic domain. J. Cell Biol..

[27] Corona J.C., Duchen M.R. (2016). PPARγ as a therapeutic target to rescue mitochondrial function in neurological disease. Free Radic. Biol. Med..

[28] Kubota N., Terauchi Y., Miki H., Tamemoto H., Yamauchi T., Komeda K., Satoh S., Nakano R., Ishii C., Sugiyama T. (1999). PPAR gamma mediates high-fat diet-induced adipocyte hypertrophy and insulin resistance. Mol. Cell.

[29] Tontonoz P., Hu E., Spiegelman B.M. (1994). Stimulation of adipogenesis in fibroblasts by PPAR gamma 2, a lipid-activated transcription factor. Cell.

[30] Karunakaran U., Elumalai S., Moon J.-S., Won K.-C. (2021). CD36 Signal Transduction in Metabolic Diseases: Novel Insights and Therapeutic Targeting. Cells.

[31] Cai L., Wang Z., Ji A., Meyer J.M., van der Westhuyzen D.R. (2012). Scavenger receptor CD36 expression contributes to adipose tissue inflammation and cell death in diet-induced obesity. PLoS One.

[32] Al-Rashed F., Haddad D., Al Madhoun A., Sindhu S., Jacob T., Kochumon S., Obeid L.M., Al-Mulla F., Hannun Y.A., Ahmad R. (2023). ACSL1 is a key regulator of inflammatory and macrophage foaming induced by short-term palmitate exposure or acute high-fat feeding. iScience.

[33] Chen Y., Zhang J., Cui W., Silverstein R.L. (2022). CD36, a signaling receptor and fatty acid transporter that regulates immune cell metabolism and fate. J. Exp. Med..

[34] Miao Y., Zhang C., Yang L., Zeng X., Hu Y., Xue X., Dai Y., Wei Z. (2022). The activation of PPARγ enhances Treg responses through up-regulating CD36/CPT1-mediated fatty acid oxidation and subsequent N-glycan branching of TβRII/IL-2Rα. Cell Commun. Signal..

[35] Almeida P.E., Roque N.R., Magalhães K.G., Mattos K.A., Teixeira L., Maya-Monteiro C., Almeida C.J., Castro-Faria-Neto H.C., Ryffel B., Quesniaux V.F.J., Bozza P.T. (2014). Differential TLR2 downstream signaling regulates lipid metabolism and cytokine production triggered by Mycobacterium bovis BCG infection. Biochim. Biophys. Acta.

[36] Almeida P.E., Silva A.R., Maya-Monteiro C.M., Töröcsik D., D'Avila H., Dezsö B., Magalhães K.G., Castro-Faria-Neto H.C., Nagy L., Bozza P.T. (2009). Mycobacterium bovis bacillus Calmette-Guérin infection induces TLR2-dependent peroxisome proliferator-activated receptor gamma expression and activation: functions in inflammation, lipid metabolism, and pathogenesis. J. Immunol..

[37] Li Z., Luo L., Yu W., Li P., Ou D., Liu J., Ma H., Sun Q., Liang A., Huang C. (2022). PPARγ phase separates with RXRα at PPREs to regulate target gene expression. Cell Discov..

[38] Suzuki K., Suda G.A.-O., Yamamoto Y., Furuya K., Baba M., Nakamura A., Miyoshi H., Kimura M., Maehara O., Yamada R. (2021). Tenofovir-disoproxil-fumarate modulates lipid metabolism via hepatic CD36/PPAR-alpha activation in hepatitis B virus infection. J. Gastroenterol..

[39] Wu L., Wang K., Wang W., Wen Z., Wang P., Liu L., Wang D.W. (2018). Glucagon-like peptide-1 ameliorates cardiac lipotoxicity in diabetic cardiomyopathy via the PPARα pathway. Aging Cell.

[40] Al-Rashed F., Haddad D., Al Madhoun A., Sindhu S., Jacob T., Kochumon S., Obeid L.M., Al-Mulla F., Hannun Y.A., Ahmad R. (2023). ACSL1 is a key regulator of inflammatory and macrophage foaming induced by short-term palmitate exposure or acute high-fat feeding. iScience.

[41] Luo Y., Tanigawa K., Kawashima A., Ishido Y., Ishii N., Suzuki K. (2020). The function of peroxisome proliferator-activated receptors PPAR-γ and PPAR-δ in Mycobacterium leprae-induced foam cell formation in host macrophages. PLoS Neglected Trop. Dis..

[42] Deng T., Lyon C.J., Minze L.J., Lin J., Zou J., Liu J.Z., Ren Y., Yin Z., Hamilton D.J., Reardon P.R. (2013). Class II major histocompatibility complex plays an essential role in obesity-induced adipose inflammation. Cell Metabol..

[43] Józefowski S., Sobota A., Pawłowski A., Pawłowski A., Kwiatkowska K. (2011). Mycobacterium tuberculosis lipoarabinomannan enhances LPS-induced TNF-α production and inhibits NO secretion by engaging scavenger receptors. Microb. Pathog..

[44] Tailleux L., Schwartz O., Herrmann J.-L., Pivert E., Jackson M., Amara A., Legres L., Dreher D., Nicod L.P., Gluckman J.C. (2003). DC-SIGN is the major Mycobacterium tuberculosis receptor on human dendritic cells. J. Exp. Med..

[45] Chatterjee D., Lowell K., Rivoire B., McNeil M.R., Brennan P.J. (1992). Lipoarabinomannan of Mycobacterium tuberculosis. Capping with mannosyl residues in some strains. J. Biol. Chem..

[46] Means T.K., Wang S., Lien E., Yoshimura A., Golenbock D.T., Fenton M.J. (1999). Human toll-like receptors mediate cellular activation by Mycobacterium tuberculosis. J. Immunol..

[47] Quesniaux V.J., Nicolle D.M., Torres D., Kremer L., Guérardel Y., Nigou J., Puzo G., Erard F., Ryffel B. (2004). Toll-like receptor 2 (TLR2)-dependent-positive and TLR2-independent-negative regulation of proinflammatory cytokines by mycobacterial lipomannans. J. Immunol..

[48] Kang P.B., Azad A.K., Torrelles J.B., Kaufman T.M., Beharka A., Tibesar E., DesJardin L.E., Schlesinger L.S. (2005). The human macrophage mannose receptor directs Mycobacterium tuberculosis lipoarabinomannan-mediated phagosome biogenesis. J. Exp. Med..

[49] Vignal C., Guérardel Y., Kremer L., Masson M., Legrand D., Mazurier J., Elass E. (2003). Lipomannans, but not lipoarabinomannans, purified from Mycobacterium chelonae and Mycobacterium kansasii induce TNF-alpha and IL-8 secretion by a CD14-toll-like receptor 2-dependent mechanism. J. Immunol..

[50] Al-Rashed F., Ahmad Z., Snider A.J., Thomas R., Kochumon S., Melhem M., Sindhu S., Obeid L.M., Al-Mulla F., Hannun Y.A., Ahmad R. (2021). Ceramide kinase regulates TNF-α-induced immune responses in human monocytic cells. Sci. Rep..

[51] Haider M.A.-O.X., Albaqsumi Z., Al-Mulla F.A.-O., Ahmad R.A.-O., Al-Rashed F.A.-O. (2022). SOCS3 Regulates Dectin-2-Induced Inflammation in PBMCs of Diabetic Patients. Cells.

