# Supplementary material for: PPARdelta: A key modulator in the pathogenesis of diabetes mellitus and Mycobacterium tuberculosis co-morbidity
Source: iScience. 2024 May 22;27(7):110046. doi: 10.1016/j.isci.2024.110046 (PMC11233913; doi:10.1016/j.isci.2024.110046)
Supplement: Document S1. Figures S1 and S2 and Tables S1 and S2 [file mmc1.pdf]

iScience, Volume *27*

Supplemental information

PPARdelta: A key modulator in the pathogenesis of diabetes mellitus and *Mycobacterium tuberculosis* co-morbidity

Halemah AlSaeed, Mohammed J.A. Haider, Fawaz Alzaid, Fahd Al-Mulla, Rasheed Ahmad, and Fatema Al-Rashed

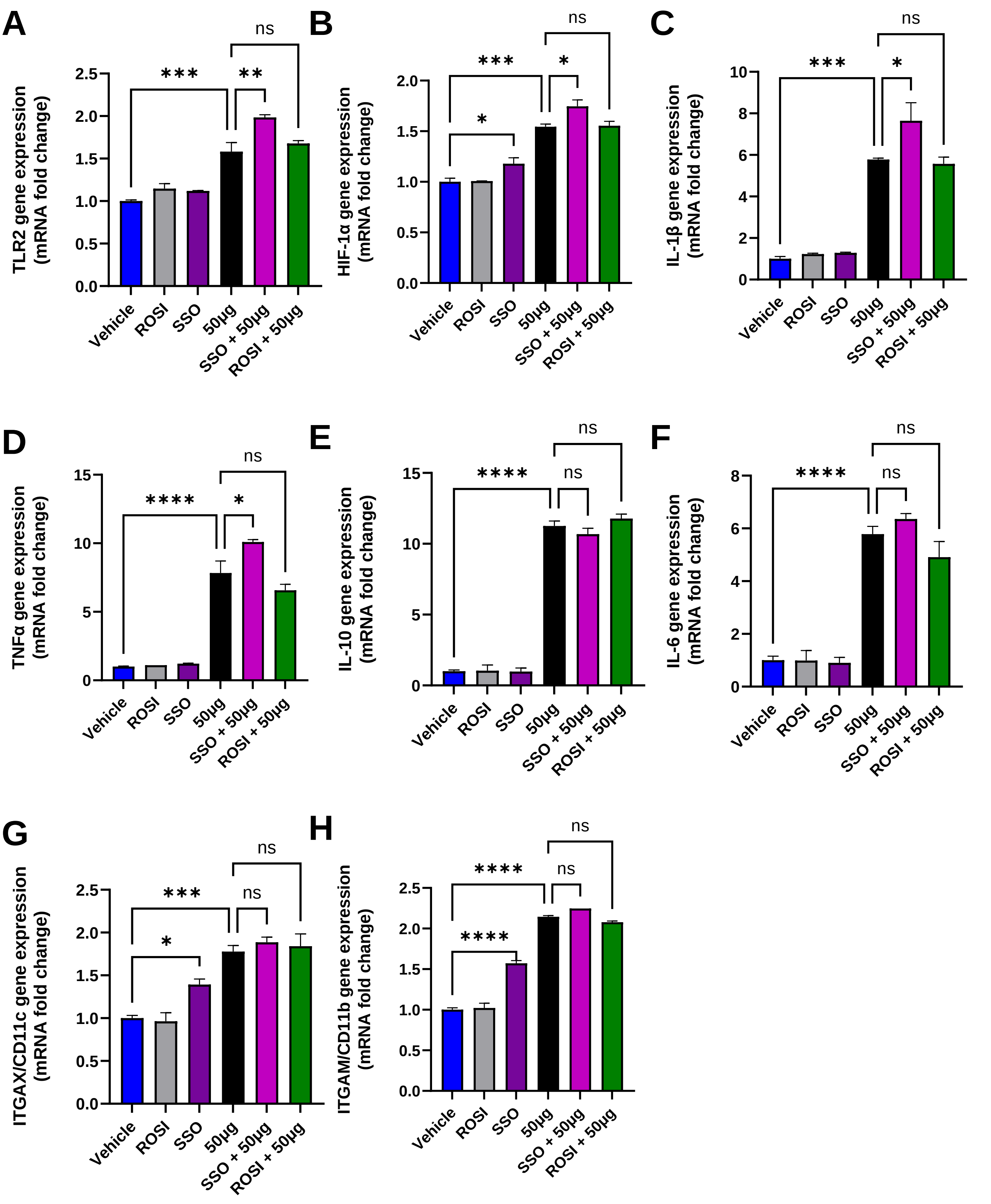

Supplementary Figure S1. The inhibition of CD36 receptor activity augmented the inflammatory response induced by HKMT. Related to Figure 2. For stimulation studies, cells were pre-treated with CD36 inhibitor SSO (250µM), or PPARγ agonist rosiglitazone (1µM), for 60 min and then stimulated with 50µg HKMT.(A) Gene expression of TLR2 determined by qRT-PCR. (B) Gene expression of HIF-1α determined by qRT-PCR. (C) Gene expression of IL-1β determined by qRT-PCR. (D) Gene expression of TNFα determined by qRT-PCR. (E) Gene expression of IL-10 determined by qRT-PCR. (F) Gene expression of IL-6 determined by qRT-PCR. (G) Gene expression of ITGAX/CD11c determined by qRT-PCR. (H) Gene expression of ITGAM/CD11b determined by qRT-PCR. Data are presented as mean ± SEM values (n=3-4) and compared between groups using one-way ANOVA with Tukey's multiple comparisons test. *$p\leq0.05$, **$p\leq0.01$, ***$p\leq0.001$, ****$p\leq0.0001$.

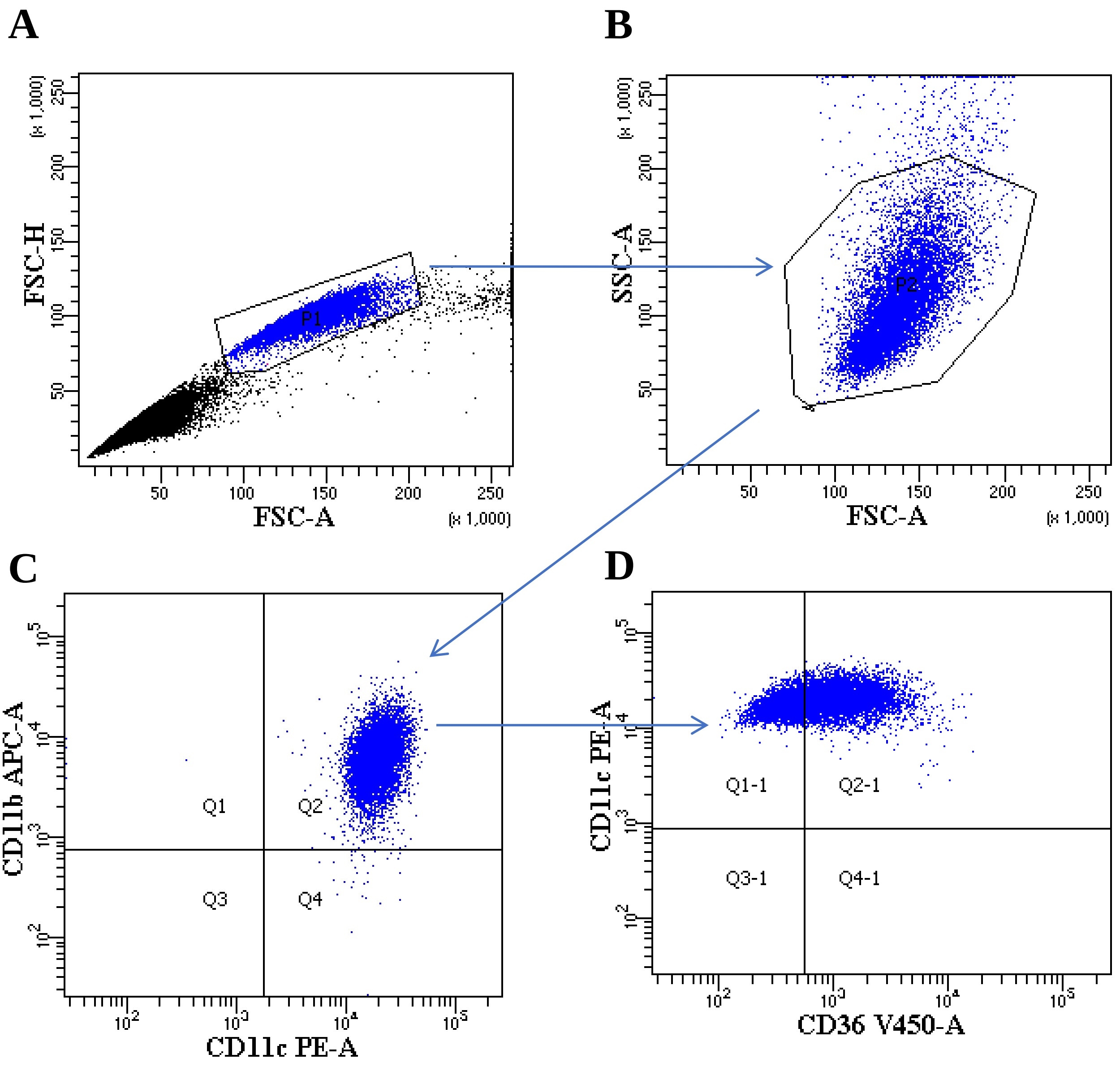

Supplementary Figure S2. Monocytes gating strategy for phenotypic characterization. Related to STAR Methods. (A-B) Single cells were gated based on their forward scatter height and area, (C)and monocytes within the single cell gate were identified as CD11B^{+}CD11c^{+} cells.(D) CD11b^{+}CD11c^{+}CD36^{+} cells were then identified within this gate, and their expression was assessed by median fluorescence intensity (MFI).

Supplementary Table 1. Patient characteristics . Related to Figure 5 and Figure 6

Characteristic	Lean ND<br>n=10 (3M/7F)	Obese ND<br>n = 11 (6M/5F)	Obese D<br>n = 10 (3M/7F)	p value
Age (years)	42 ± 8.1	44.2 ± 11.5	48 ± 12.5	0.1158
Weight (kg)	62.9 ± 11.8	86.8 ± 18.6	89.1 ± 12.5	0.0006ΦΔ
Height (cm)	1.6 ± 0.12	1.6 ± 0.08	1.69 ± 0.06	0.6821
BMI (kg/m2)	22.8 ± 2.3	30.4 ± 6.2	31.1 ± 3.4	0.0003ΦΔ
Waist circumference(inch)	81.3 ± 12.3	103.5 ± 17.9	102.5 ± 8.12	0.0038ΦΔ
Hip circumference (inch)	98.7 ± 6.8	107.0 ± 12.4	114.8 ± 10.8	0.0184Δ
Fat weight (kg)	24.5 ± 12.4	34.4 ± 7.4	36.9 ± 5.0	0.0007ΦΔ

Data presented in mean ± SD. Φ Lean ND vs OB ND, Δ Lean ND vs OB D.

Supplementary Table 2. Patient bloodwork . Related to Figure 5 and Figure 6

Blood work	Lean ND<br>n=10 (3M/7F)	Obese ND<br>n = 11 (6M/5F)	Obese D<br>n = 10 (3M/7F)	p value
Triglycerides (mmol/L)	0.63 ± 0.23	1.4 ± 0.86	1.5 ± 0.68	$0.0116^{\Phi\Delta}$
Total cholesterol (mmol/L)	5.3 ± 1.1	4.8 ± 0.77	5.0 ± 0.79	0.4564
HDL cholesterol (mmol/L)	1.6 ± 0.5	1.1 ± 0.14	1.2 ± 0.31	$0.0051^{\Phi\Delta}$
Fasting glucose (mmol/L)	4.9 ± 0.6	5.2 ± 0.48	7.8 ± 1.6	$<0.0001^{\Delta\psi}$
Insulin Con. (mu/L)	9.7 ± 8.2	5.1 ± 1.6	59.3 ± 40.2	$0.0016^{\Delta\psi}$
HOMA-IR	3 ± 2.8	3.4 ± 3.6	23.1 ± 4.4	$<0.0001^{\Delta\psi}$
HbA1c (%)	5.66 ± 0.4	5.5 ± 0.48	7.4 ± 1.5	$0.0002^{\Delta\psi}$
CRP (pg/mL)	1.9 ± 1.1	3.9 ± 5.6	7.4 ± 2.8	0.0141^{Δ}

Data presented in mean ± SD. Φ Lean ND vs OB ND, Δ Lean ND vs OB D, ψ OB ND vs OB D.
